# Role of Intestinal Microbiota in the Bioavailability and Physiological Functions of Dietary Polyphenols

**DOI:** 10.3390/molecules24020370

**Published:** 2019-01-21

**Authors:** Kyuichi Kawabata, Yasukiyo Yoshioka, Junji Terao

**Affiliations:** Faculty of Clinical Nutrition and Dietetics, Konan Women’s University, 6-2-23 Morikita-machi, Higashinada-ku, Kobe City, Hyogo 658-0001, Japan; kyuichi@konan-wu.ac.jp (K.K.); y-yoshioka@konan-wu.ac.jp (Y.Y.)

**Keywords:** polyphenol, tannin, intestinal microbiota, bioavailability, microbial catabolite, physiological function, prebiotics

## Abstract

Polyphenols are categorized as plant secondary metabolites, and they have attracted much attention in relation to human health and the prevention of chronic diseases. In recent years, a considerable number of studies have been published concerning their physiological function in the digestive tract, such as their prebiotic properties and their modification of intestinal microbiota. It has also been suggested that several hydrolyzed and/or fission products, derived from the catabolism of polyphenols by intestinal bacteria, exert their physiological functions in target sites after transportation into the body. Thus, this review article focuses on the role of intestinal microbiota in the bioavailability and physiological function of dietary polyphenols. Monomeric polyphenols, such as flavonoids and oligomeric polyphenols, such as proanthocyanidins, are usually catabolized to chain fission products by intestinal bacteria in the colon. Gallic acid and ellagic acid derived from the hydrolysis of gallotannin, and ellagitannin are also subjected to intestinal catabolism. These catabolites may play a large role in the physiological functions of dietary polyphenols. They may also affect the microbiome, resulting in health promotion by the activation of short chain fatty acids (SCFA) excretion and intestinal immune function. The intestinal microbiota is a key factor in mediating the physiological functions of dietary polyphenols.

## 1. Introduction

### 1.1. Classification

Polyphenols are plant secondary metabolites ubiquitously present in many parts of the plant, including flowers, leaves, pulp, stems, and roots. They are not directly responsible for the growth and development of plants, but are necessary for the plant to survive in its environment. They are produced from primary metabolites and intermediates through unique biosynthetic pathways. In recent years, much attention has been paid to their potential role as functional food ingredients. A variety of polyphenols and their derivatives are currently expected to be used as dietary factors to prevent chronic diseases, such as diabetes, cancer, and stroke [1,2,3,4,5]. In 2013, Del Rio et al. [6] reviewed more than 500 publications focusing on the bioavailability of dietary polyphenols and their protective effects against chronic diseases.

**Figure 1:**
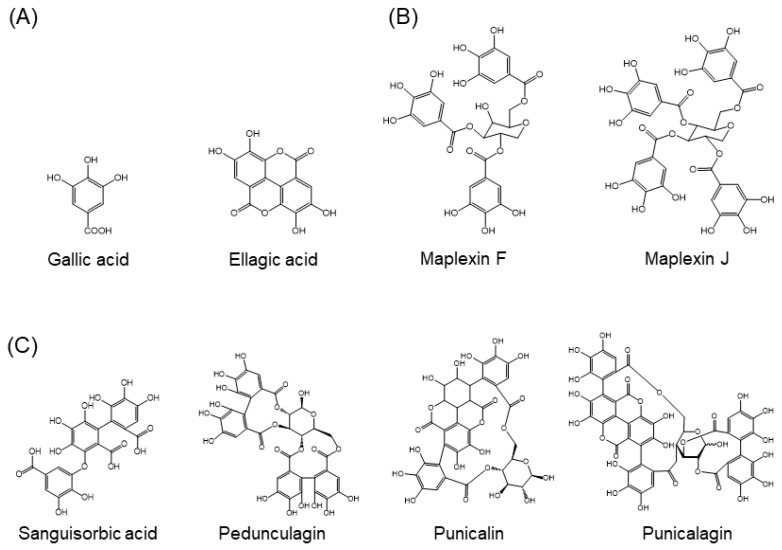
Structures of hydrolysable tannins. (**A**) Molecules making up tannins, (**B**) examples of gallotannins, (**C**) examples of ellagitannins.

**Figure 2:**
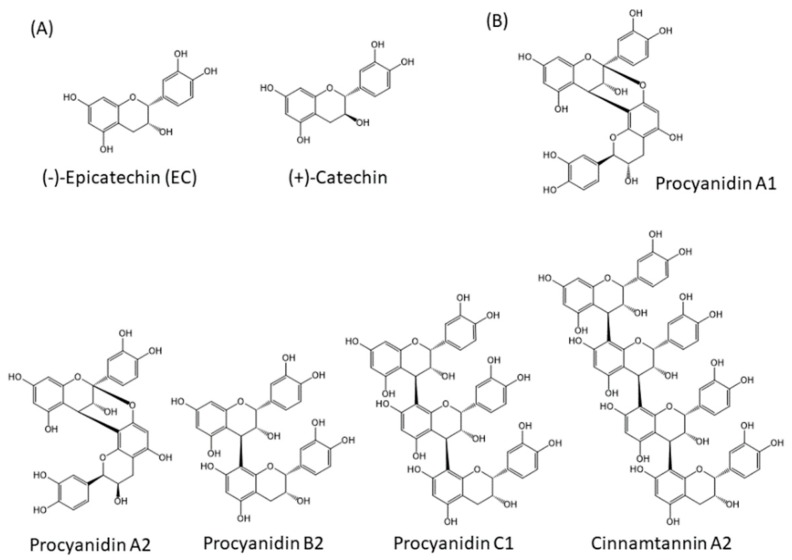
Structures of non-hydrolysable condensed tannins (proanthocyanidins). (**A**) Molecules making up proanthocyanidins, (**B**) Examples of proanthocyanidins.

**Figure 3:**
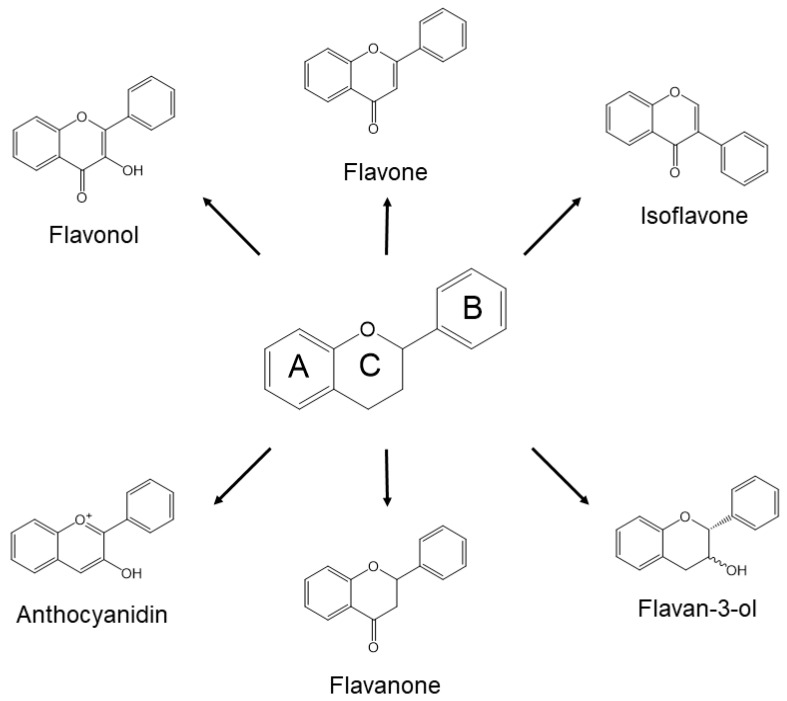
Generic structures of major flavonoids.

Approximately 8000 compounds of polyphenols are found in the plant kingdom. These can be separated into high molecular weight tannins and low molecular weight polyphenols. Tannins consist of hydrolysable tannins (gallotannins and ellagitannins, Figure 1) and non-hydrolysable, condensed tannins (proanthocyanidins, Figure 2) [7]. Low molecular weight polyphenols can be categorized into several subgroups: phenolic acid derivatives, flavonoids, lignans, stilbenes, and curcumins. For many years, large numbers of studies have been undertaken to discover the physiological function of low molecular weight polyphenols, in particular, flavonoids; carboxylic acids, such as caffeic acid and ferulic acid; and resveratrol [8]. Flavonoids are typical low molecular weight polyphenols present in plant foods, and they are characterized by a coplanar diphenylpropane structure (Figure 3). Among them, catechins (flavan-3-ols) are also present as molecular units consisting of proanthocyanidins.

### 1.2. Background of Polyphenol Research

In 1936, Bentsáth et al. [9] first reported that citrus flavonoids (hesperidin and rutin) reduced capillary fragility and permeability in human blood vessels. Thereafter, the vascular effects of plant-derived flavonoids were gradually explored [10]. In 1993, an epidemiological study in a Dutch population first revealed that the high intake of flavonoids (mainly flavonol-type flavonoids) correlates with a decrease in death due to atherosclerotic vascular disease [11]. This study encouraged researchers investigating dietary flavonoids and triggered human intervention studies of their bioavailability and bio-efficacy [12].

Polyphenols, including flavonoids, can act as antioxidants because they possess electron-donating phenolic groups in their structures. Therefore, several studies have investigated their antioxidant function in the prevention of oxidative stress-related cellular and extracellular damage. Antioxidant function has long been suggested to have a major role in promoting vascular health for the prevention of atherosclerosis [13]. Nevertheless, the bioavailability of flavonoids is significantly lower than the bioavailability of antioxidant vitamins and pro-vitamins (vitamin E, vitamin C, and carotenoids). Hollman et al. [14] suggested that antioxidant function may not explain the major vascular effects of dietary flavonoids. The cellular targets of flavonoids may be kinases involved in signal transduction pathways (Raf, Fyn, MEK, and PI3K) [15]. The resulting modulation of these pathways seems to be a major component of the physiological role of flavonoids after their absorption into the body [16]. They are able to act as modulators of cellular signaling pathways, provided that they are efficiently absorbed into the body and accumulate in target cells or target fluids [10,17]. The bioavailability of dietary flavonoids is, in general, very low. Therefore, they are mostly excreted into feces after transportation into the gut. Furthermore, high molecular weight tannins are scarcely absorbed in their original form because of their relatively large molecular size. Investigations into their physiological function has long been neglected or limited to their role in the digestive tract. The antioxidant effects of dietary polyphenols may only be exerted in the digestive tract, because of their high concentration in the gut. However, recent studies on the relationship between intestinal microbiota and dietary polyphenols have provoked new ideas on the physiological function of non-absorbable polyphenols, such as non-hydrolysable and hydrolysable tannins [18].

### 1.3. Interest in the Actions of Intestinal Microbiota

The gut microbiota is strongly associated with the occurrence of obesity by increasing the capacity for energy harvest [19]. It is, therefore, likely that an alteration of the intestinal microbiota may contribute to the improvement of human health. In fact, recent studies have demonstrated that non-absorbable polyphenols act in the intestine to alter the gut microbial community, resulting in lower systemic inflammation and improved metabolic outcomes [20,21,22]. Polyphenols may also exert beneficial effects on microbiota by acting as prebiotics [23].

In addition, a large new area of study involves investigating the effect of the gut microbiota on the brain and behavioral traits [24,25]. However, the association between behavior and the action of dietary polyphenols in the digestive tract is not well understood. On the other hand, the bioconversion of dietary polyphenols by gut microbiota has recently been shown to affect the nutritional phenotype of humans [26]. Enterobacteria-dependent catabolites of polyphenols may exert their effects in the body by transferring into the blood circulation. For example, urolithin or its glucuronide conjugate, an ellagitannin catabolized by intestinal bacteria, has been reported to increase muscle function in rodents [27], inhibit metastasis of cancer cell lines [28], and protect the impairment of cardiomyocytes [29]. Intestinal polyphenol catabolites may also exert their effects within the intestinal tract and intestinal walls. These may include inhibitory effects on colorectal cancer and suppressive effects on inflammatory bowel diseases [30,31,32,33].

The purpose of this review is to focus on the physiological effects of non-absorbable monomeric and polymeric polyphenols on the intestinal microbiota. Colonic bacteria-dependent catabolic pathways, bioavailability, and the function of their catabolites in the digestive tract and circulatory system is also discussed. Finally, we will discuss the mechanism by which plant polyphenols contribute to human health through the intestinal microbiota. In this paper, we will review flavonoids, tannins, curcumins, and resveratrol, but not isoflavones.

## 2. Hydrolysis and Absorption of Polyphenols in the Stomach and Small Intestine

### 2.1. Stability of Polyphenols in the Stomach

In general, low molecular weight polyphenols are partially absorbed into the body directly or after phase II enzyme-dependent metabolic conversion in small intestinal cells, as described above [34]. High molecular weight tannins and even low molecular weight polyphenols are transported into the large intestine in their original form. They are then excreted with feces without intestinal absorption, or they are catabolized by enterobacteria [35,36]. Therefore, the stomach is the minor location of absorption of polyphenols. In fact, low molecular weight polyphenols are stable, and glucose-bound polyphenols are only partially hydrolyzed, despite the low pH in the stomach [37]. It should be noted that anthocyanins (anthocyanidin glucosides) can be absorbed from the stomach via an active transport pathway involving bilitranslocase, an organic anion membrane carrier [38]. The stability of tannins in the stomach is similar to that of low molecular weight polyphenols [39]. However, the data for proanthocyanidins are more conflicting. An in vitro study suggests that procyanidins are hydrolyzed to bioavailable flavan-3-ol monomers [40], but an in vivo study claims a lack of depolymerization [41].

### 2.2. Intestinal Absorption of Sugar-Bound Polyphenols

**Figure 4:**
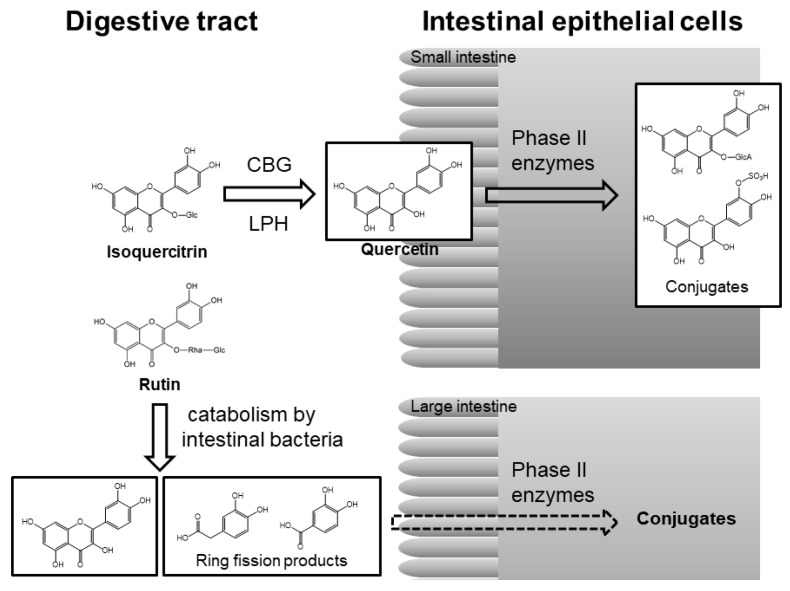
Pathway of absorption and metabolism of isoquercitrin (quercetin 3-*O*-glucoside) and rutin (quercetin 3-*O*-β-rutinoside) in the digestive system [34,46,48]. CBG, cytosolic β-glucosidase; LPH, lactose phlorizin hydrolase; Glc, glucose; Rha, rhamnose; GlcA, glucuronic acid.

In the small intestine, glucose-bound polyphenols are deglucosylated to aglycone by lactase-phlorizin hydrolase (LPH) or cytosolic β-glucosidase (CBG) of intestinal epithelial cells and then converted into *O*-glucuronide/*O*-sulfate conjugates by phase II enzymes in intestinal cells [34]. These conjugated metabolites are transported into the liver, where they are further metabolized, after which they either enter into the blood circulation or return to the digestive tract through the enterohepatic circulation (Figure 4). The total concentration of conjugated metabolites may reach micromolar levels within a few hours and then almost disappear after 24 h [42].

Other glycosides that are bound to sugars other than glucose are not substrates for these hydrolytic enzymes that release aglycone in the small intestine [34]. The time to reach C_max_ (the compound’s maximum concentration in the blood) is significantly longer after rutin (quercetin-3-*O*-β-rutinoside) supplementation than after quercetin aglycone supplementation [43]. In contrast to quercetin aglycone and its glucosides, rutin is barely absorbed in the small intestine [44,45]. Most dietary rutin enters into the large intestine, where the colonic microbiota liberates quercetin aglycone from rutin. Quercetin aglycone can then be absorbed or further degraded to produce various ring-fission products by the action of enterobacteria (Figure 4) [46,47].

### 2.3. Intestinal Absorption of Monomeric Epicatechin and Its Related Monomeric Flavan-3-ols

In the case of flavan-3-ols, monomeric epicatechin is partly absorbed in the small intestine in its original form and is then subjected to glucuronidation, sulfation and/or *O*-methylation [49]. Natsume et al. [48] identified (−)-epicatechin-3′-*O*-glucuronide, 4′-*O*-methyl-(−)-epicatechin-3′-*O*-glucuronide, and 4′-*O*-methyl-(−)-epicatechin-5 or 7-*O*-glucuronide as metabolites of (−)-epicatechin in human urine. Ottaviani et al. [50] demonstrated that 82 ± 5% of ingested (−)-epicatechin is absorbed, and more than 20 different metabolites are present in human plasma after ingestion of [2-^14^C](−)-epicatechin. They also suggested that the gut microbiota is a key driver of (−)-epicatechin metabolism, because the concentration of its metabolites in plasma shows a biphasic pattern as a function of time. In the case of green tea flavan-3-ols, overall flavan-3-ol metabolite excretion is estimated to be 8.1% of intake [51]. Interestingly, un-metabolized (−)-epigallocatechin-3-gallate and (−)-epicatechin-3-gallate are also detected in the plasma, together with *O*-methylated, *O*-sulfated, and *O*-glucuronide conjugates of (−)-epicatechin and (−)-epigallocatechin [52].

### 2.4. Intestinal Absorption of Oligomeric Procyanidins

It is generally accepted that procyanidin polymers and oligomers with a degree of polymerization (DP) > 4 are not directly absorbed from the small intestine, although dimers and trimers can be detected in the plasma [53]. A-type procyanidin dimers, trimers, and tetramers can be transported across human intestinal epithelial Caco-2 cells [54]. Shoji et al. [55] found that apple procyanidins of each group, from dimers to pentamers, are present in rat plasma. Donovan et al. [39] claimed that neither monomers nor oligomers existed at detectable concentrations in vivo when grapeseed procyanidins were administered to rats. However, Serra et al. [56] reported free forms of dimers and trimers in rat plasma after oral intake of a grapeseed extract. The degree of polymerization has a major impact on the fate of procyanidins in the body, with greater degrees of polymerization showing poorer absorption through the gut barrier [57]. Baba et al. [58] found that the procyanidin dimer, B2 [epicatechin-(4-8)-epicatechin], is absorbed and a portion of B2 is degraded to monomeric epicatechin after B2 administration to rats. Interestingly, another study in rats showed that the procyanidin dimer, A1 [epicatechin-(2-*O*-7,4-8)-catechin] and the procyanidin dimer, A2 [epicatechin-(2-*O*-7, 4-8)-epicatechin] are better absorbed than the procyanidin dimer, B2 [epicatechin-(4-8)-epicatechin], although absorption of the A-type dimers was only 5–10% of monomeric epicatechin absorption [59].

### 2.5. Hydrolysis of Tannins in the Digestive Tract

A human pilot trial using gallotannin-rich mango pulp (*Mangifera indica* L. cv. Keitt) strongly suggested that gallotannins release free gallic acid in the gastrointestinal tract, indicating that gallotannins may serve as a pool of pro-gallic acid compounds that can be absorbed or can undergo microbial metabolism [60]. In humans, ellagic acid can be released by the hydrolysis of ellagitannins after the consumption of pomegranate juice. This is facilitated by physiological pH and/or gut microbiota actions [61]. A study of ileostomists consuming raspberries indicated that the hydrolysis of ellagitannins occurs in the stomach or small intestine [62]. Therefore, hydrolysable tannins can be hydrolyzed in the digestive tract before absorption or microbial catabolism.

## 3. Decomposition and Metabolism by Intestinal Bacteria

The gut microbiota influences polyphenol bioavailability by modifying the structure of aglycones, glycosides, and conjugates, such as *O*-glucuronides and *O*-sulfates. A large proportion of *O*-glycosides are converted into their corresponding aglycones by the acidic conditions of the stomach, by mucosal enzymes in the small intestine, or by enzymes in the gut microbiota. Aglycones are subjected to the activity of phase II enzymes during the absorption process and after their delivery to the liver, resulting in *O*-glucuronides and/or *O*-sulfates. These polyphenol conjugates are then excreted in the bile and re-enter the intestinal tract. In addition to enzymatic activity to deconjugate *O*-glycosides and *O*-glucuronides, gut microbes have catabolic capacity to perform carbon-carbon cleavage of heterocyclic and aromatic rings, dehydroxylation, decarboxylation, and hydrogenation of alkene moieties. These catabolic transformations follow a procedure of reactions that conform to the basic principles of chemistry. In this section, we outline the mechanism of the gut microbial transformation of major dietary polyphenols.

### 3.1. Flavonoid Quercetin

**Figure 5:**
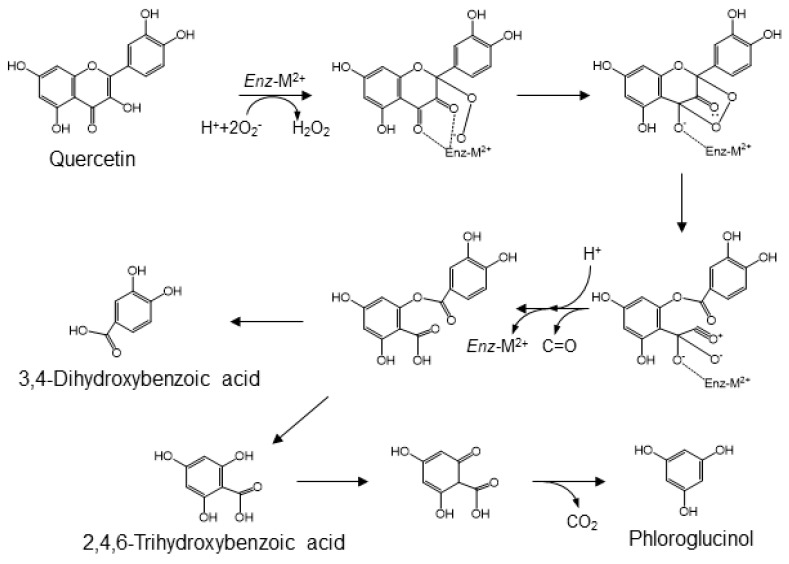
A mechanism for quercetinase-mediated conversion of quercetin into protocatechuic acid, 2,4,6-trihydroxybenzoic acid and phloroglucinol [64,65,66,67].

Quercetin, one of the major flavonols, is ubiquitously distributed in plants. It is mainly present as *O*-glycosides in various fruits and vegetables. The bacterial catabolites of rutin have been identified as 3,4-dihydroxyphenylacetic acid, 3-hydroxyphenylacetic acid, and 3-(3-hydroxyphenyl)propionic acid [44,47,63]. Several studies on the catabolism of quercetin or its glycosides by fecal microbiota have demonstrated an alternative conversion that generates 3,4-dihydroxybenzoic acid (i.e., protocatechuic acid) and 2,4,6-trihydroxybenzoic acid as the major catabolites (Figure 5) [64,65,66,67]. These catabolites are then further transformed into their *O*-methyl derivatives [68]. The enzymatic cleavage of quercetin to 3,4-dihydroxybenzoic acid is mediated by fungal and bacterial quercetin dioxygenases, known as quercetinases [69,70]. These enzymes contain divalent metal ions, such as iron, copper, or manganese, in their catalytic domain [71]. A reaction involving the chelation of oxygen atoms at positions 3 and 4 and the generation of superoxide or peroxyl radical species is proposed as the mechanism for producing 3,4-dihydroxybenzoic acid [69,72]. That is, quercetin degradation by quercetinase is initiated by chelation of the 4-keto/2,3-enol moiety with a divalent cation, resulting in the 3,4-diketo tautomer. The metal ion-bound flavonol complex places the oxygen atoms at position 2 via semi-quinone radicals, derived from phenoxy radicals. Arrangement of the endo-peroxide, based on the conversion of the 3-keto group into an acylium group, results in an ester, 2-protocatechuoyl-phloroglucinol carboxylic acid, linking the two aromatic rings and the release of carbon monoxide. While quercetin dioxygenases yield 2-protocatechuoyl-phloroglucinol carboxylic acid after incubation of quercetin with the microbiota, no catabolite of quercetin formed by hydrolytic cleavage of the ester, that is, 3,4-dihydroxybenzoic acid or 2,4,6-trihydroxybenzoic acid, is found in either animals or humans. On the other hand, there are no reports of 2,4,6-trihydroxybenzoic acid as a catabolite of quercetin. This may be because a large portion of 2,4,6-trihydroxybenzoic acid may be modified to phloroglucinol, as the known gut microbial catabolite of quercetin [73].

Quercetinase- and other dioxygenase-mediated catabolic conversions depend on oxygen molecules. However, these microbial reactions can occur in the anoxic environment of the colon. The gastrointestinal mucosa separates the highly vascularized and oxygen-rich epithelium from the anoxic gut lumen, and the colon, which is relatively anoxic compared with the small intestine, is helpful for intestinal bacteria to form the more complex community [74,75]. Blood vessels in the sub-epithelial tissue of the intestine accelerate efficient nutrient uptake and supply oxygen to the epithelium and then, indirectly, to the gut lumen by diffusion. The resulting steep oxygen gradient across the epithelial cell layer affords stress on the epithelium and on the absolute anaerobic micro-organisms in the gut lumen. Facultative anaerobic micro-organisms may survive and thrive at this oxic/anoxic interface without interference from absolute anaerobes. *Bacillus subtilis* has been documented as an aerobic soil microbe that can be a facultative anaerobe [76]. Moreover, its isolation and behavior in the human intestinal tract has led to its recognition as a normal gut commensal bacterium in humans [77]. Quercetinase from *B. subtilis* has been overexpressed in *E. coli*, isolated, and biochemically characterized [70,78]. Based on these studies, *B. subtilis* is proposed to be a quercetinase-dependent producer of protocatechuic acid from dietary quercetin in the human gut. As a major gut microbial metabolite of quercetin, protocatechuic acid contributes to the health effects of fruits and vegetables rich in these flavonoids.

### 3.2. Proanthocyanidin

**Figure 6:**
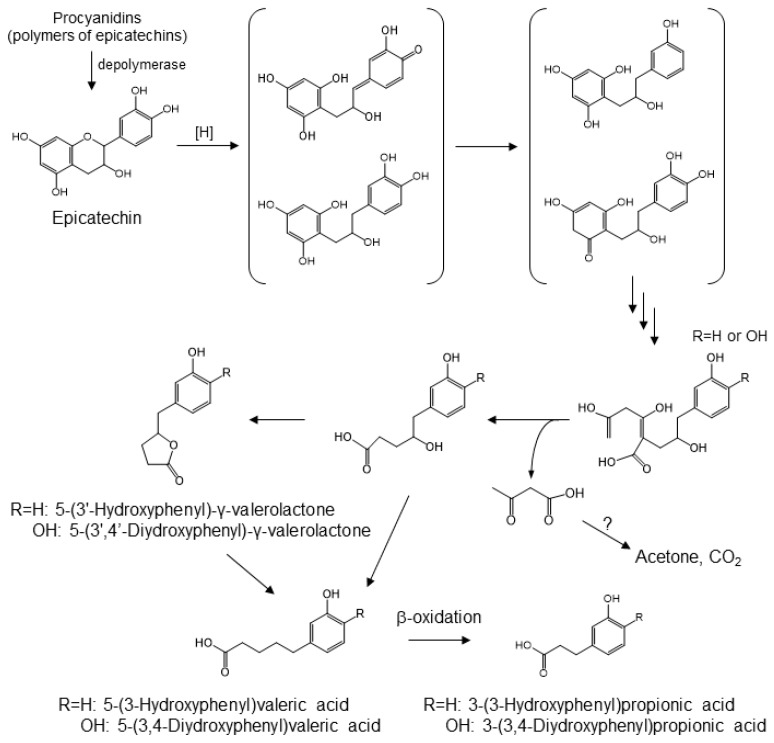
Gut microbial conversion of procyanidin [81].

Dietary proanthocyanidins, which are formed by the condensation of single or multi-component of flavan-3-ols, are found in apples, chocolate, and grapes [79]. While monomeric, dimeric, and trimeric catechins are absorbed, to some extent, from the small intestine, larger oligomers have very poor bioavailability [80]. It has been demonstrated that proanthocyanidins undergo partial acid-catalyzed cleavage into monomeric flavan-3-ol units in the gastric environment [40]. The microbial catabolites of monomeric and oligomeric catechins in the large intestine appear to be 3-hydroxyphenylacetic acid, 3,4-dihydroxyphenylacetic acid, 3-(3-hydroxyphenyl)propionic acid, and 5-(3′-hydroxyphenyl)-γ-valerolactone [81]. The latter catabolite is the result of A-ring cleavages by two consecutive reverse Claisen reactions (Figure 6). Cleavage of the C-ring of a methylated quinone intermediate prepares the molecule for retro-Claisen cleavage. There are two possible mechanisms for the catabolism of catechin. One possible mechanism is a reduction of the quinone carbonyl, yielding the corresponding *p*-hydroxy catabolite, which is then subjected to dehydroxylation. The other one is that the carbonyl quinone hydride attacks at the methylated carbon, yielding 3,4-dihydroxyphenyl catabolites, which then eventually give rise to 5-(3′,4′-dihydroxyphenyl)-γ-valerolactone. The latter compound has been reported to be a product of gut microbial catabolism [82,83,84,85,86,87].

### 3.3. Anthocyanidins

**Figure 7:**
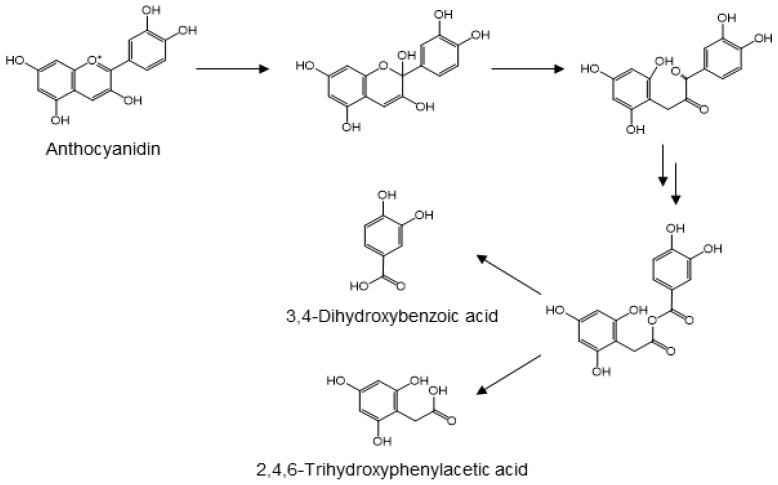
Proposed mechanism for the microbial conversion of anthocyanidin into protocatechuic acid and 2,4,6-trihydroxyphenylacetic acid [96].

Anthocyanidins, a subclass of flavonoid plant pigments, are responsible for red, purple, and blue colors of flower petals, vegetables, and fruits. Anthocyanins are the glycosylated forms of their corresponding aglycones. While the glycosides are bioavailable, they are susceptible to hydrolytic conversion into their corresponding anthocyanidins [88,89,90,91,92]. The glycosides and aglycones are both found as glucuronides in the urine of animals and humans [93,94]. The anthocyanidins are frequently metabolized into protocatechuic acid [95]. Using cyanidin as the prototypic anthocyanidin, its metabolism is initiated by cleavage of the heterocyclic flavylium ring at neutral or slightly basic conditions of the small intestine. Subsequent attack of the flavylium carbon at position 2 produces an unstable hemi-ketal that rapidly forms a ketone. Through keto-enol tautomerism of the neighboring enol functionality, the resulting α-diketo group is cleaved by gut microbiota to form protocatechuic acid and 2,4,6-trihydroxyphenylacetic acid (Figure 7). Although the cleavage reaction mechanism remains to be elucidated, it seems reasonable to propose that the C-C cleavage involves the attack of either carbonyl by a peroxyl anion species, similar to the initial step in the dioxygenase-mediated conversion of α-ketoglutarate into succinate [96]. Insertion of the resulting alkoxy oxygen between the original carbonyl carbons yields an anhydride, which forms two phenolic acids upon hydrolysis (Figure 7). This proposed mechanism is also similar to that of oxidative conversion of benzoins into benzoic acids [97]. After absorption from the intestinal tract and hepatic phase II metabolism, these phenolic acids are frequently detected in the urine, either in their original form or as their *O*-glucuronide or *O*-methyl derivatives [98,99,100].

### 3.4. Curcumin

**Figure 8:**
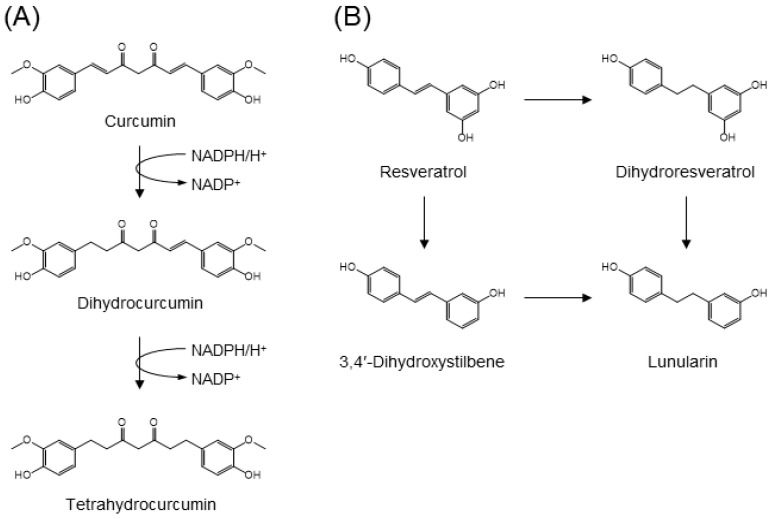
Gut microbial metabolism of (**A**) curcumin by curcumin reductase CurA and (**B**) resveratrol into lunularin [101,102,104].

In animals and humans, curcumin, a yellow pigment from turmeric, is catabolized to its hydrogenated (dihydro-, tetrahydro-, hexahydro-, and octahydro-), desmethyl, *O*-glucuronide, and *O*-sulfate metabolites [101,102]. *E. coli* NADPH-dependent reductase, the enzyme responsible for the stepwise reduction of curcumin, has been isolated from human feces (Figure 8A) [103]. While the enzyme, NADPH-dependent curcumin/dihydrocurcumin reductase (CurA), catalyzes curcumin selectively, it does not reduce tetrahydrocurcumin to yield corresponding secondary alcohols. CurA has been identified as a member of the medium-chain dehydrogenase/reductase superfamily [103].

### 3.5. Resveratrol

Resveratrol is a stilbene-type polyphenol characteristically present in red wine and grapes. The main gut microbial catabolites of resveratrol are dihydroresveratrol and the *m*-deoxy metabolites of both resveratrol and dihydroresveratrol (Figure 8B) [104]. Using a panel of gut microbes, *Slackia equolifaciens* and *Adlercreutzia equolifaciens* were identified as dihydroresveratrol producers. Upon incubation of resveratrol with fecal samples, both dihydroresveratrol and 3,4′-dihydroxystilbene were observed as intermediates in the formation of lunularin [104].

### 3.6. Ellagitannin

**Figure 9:**
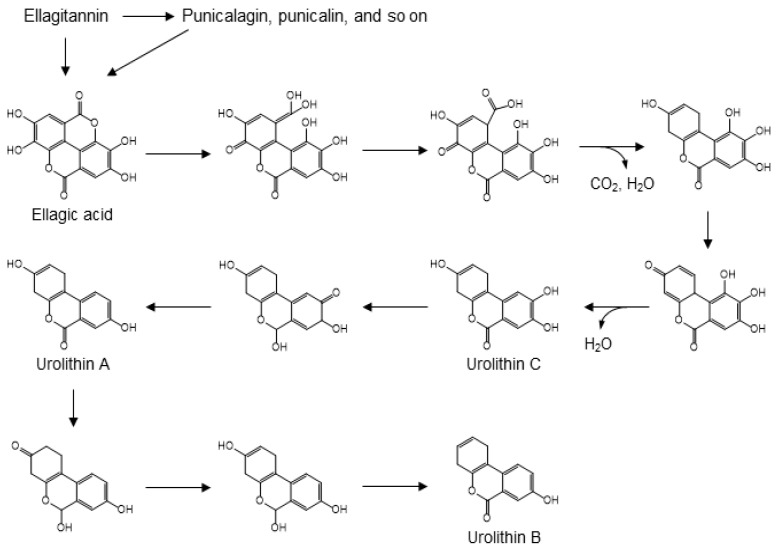
Gut microbial conversion of ellagitannin into urolithins A, B, and C [108,109,110,111].

Ellagitannins, including ellagic acid, punicalin and punicalagin, are known to be present in pomegranates, raspberries, strawberries, and walnuts [105,106]. Acid hydrolysis of ellagitannins releases free ellagic acid [107]. The process of gut microbial conversion of ellagic acid into urolithins can be explained by the carboxyl group-driven dehydroxylation of polyphenols. Strawberries, pomegranate juice, and walnuts are good sources of dietary ellagic acid, which is formed by C-C coupling of two molecules of gallic acid, followed by intramolecular condensation to form a di-lactone [108,109,110,111]. Urolithins are, arguably, the main urinary biomarkers of nut consumption [112]. In the catabolic pathway from ellagic acid to urolithins, one of the two lactone moieties undergo hydrolysis and the methylated quinone tautomer of the resulting carboxylic acid is then reduced in a manner similar to the ferredoxin-mediated reduction of 4-hydroxybenzoic acid (Figure 9). This results in a semi-hydroquinone, from which the *p*-hydroxy group leaves as a water molecule following decarboxylation. Subsequent dehydroxylation can occur via reduction of quinone-methide tautomers to form urolithins A, B, and C. In the microbial pathway from ellagic acid to urolithins, only the first dehydroxylation is driven by decarboxylation. Subsequent dehydroxylations involve a step-by-step reduction of a quinone, in which keto-enol tautomerism first produces a secondary alcohol and a subsequent hydride attack of the quinone leads to the dehydration step. Analogous dehydration is found in the dehydroxylation of catechin (Figure 6), which leads to the formation of 5-(3′-hydroxyphenyl)-γ-valerolactone. In addition, the pathway described for the dehydroxylation of 4-hydroxybenzoic acid may explain the formation of 3-hydroxybenzoic acid from protocatechuic acid after C-ring cleavage of cyanidin or quercetin (Figure 5).

## 4. Physiological Functions Mediated by Intestinal Microbiota

### 4.1. Bioavailability and Action of Polyphenol Catabolites

**Figure 10:**
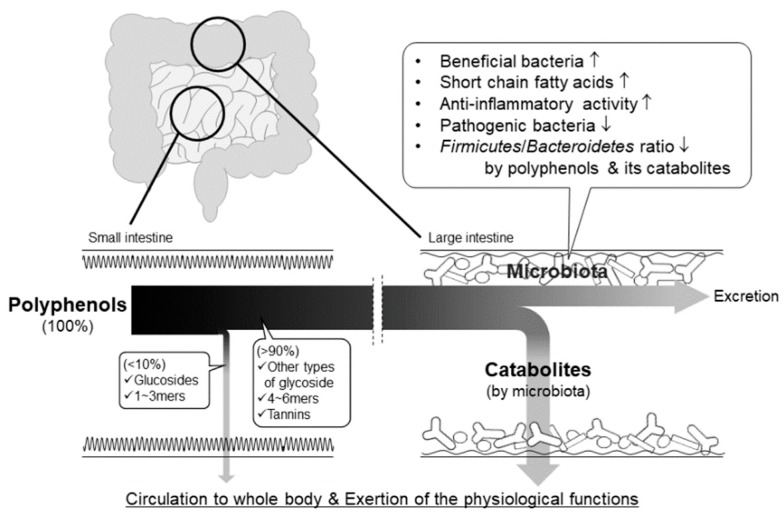
Pathways of absorption, bioconversion, and physiological action of non-absorbable polyphenols in the intestinal tract.

The total intake of dietary polyphenol at the small intestine is estimated at around 10% [113,114,115]. Hence, a large proportion of ingested polyphenols are transported to the large intestine, where they are catabolized to phenolic acids by the intestinal bacteria. Recently, much attention has been paid to the bioavailability and physiological actions of their catabolites and to the functional interaction between polyphenols and intestinal bacteria (Figure 10).

Cyanidin glucosides are mainly converted into 3,4-dihydroxybenzoic acid in the human large intestine and are then absorbed into the bloodstream [116]. In the plasma, it is estimated that 44% and 0.02% of ingested cyanidin glucoside is in the form of 3,4-dihydroxybenzoic acid and cyanidin-3-glucoside, respectively [116], suggesting that 3,4-dihydroxybenzoic acid is a key compound for the vascular effects of cyanidin glucosides (Table 1). The intake of a cranberry juice cocktail containing mainly peonidin and cyanidin glycosides, hyperoside, and quercein, increases the plasma levels of these flavonoids and phenolic acids (Table 1) [117]. This study also showed a higher concentration of 3,4-dihydroxybenzoic acid than quercetin in human plasma. Plasma antioxidant capacity, estimated by the Oxygen Radical Absorption Capacity (ORAC) assay, correlates with the plasma levels of quercetin, epicatechin, 3,4-dihydroxybenzoic acid, 4-hydroxy-3-methoxy-benzoic acid, and 3,4-dihydroxyphenylacetic acid. 3,4-Dihydroxybenzoic acid exhibits the strongest correlation with plasma ORAC activity (r = 0.440) and also correlates with plasma TAP activity (r = 0.233) and protective activity against LDL oxidation (r = 0.503). Epicatechin conjugates, including *O*-sulfate and *O*-glucuronide, in human plasma after oral intake of 207 μmol of epicatechin, showed higher C_max_ and faster T_max_ than those of their conjugated catabolites (Table 1) [50]. However, the AUC for 0–24 h of the conjugated catabolites was higher than that of the epicatechin conjugates due to a longer elimination half-life. These pharmacokinetic data show that the increase in catabolites and their conjugates in plasma occurs around 7 h after detection of the parent flavonoids, due to colonic microbiota-dependent catabolism. Additionally, the bimodal peak of the original flavonoids suggests that they may be absorbed from the large intestine. A large proportion of ingested flavonoids seem to be transported into the large intestine, where they are degraded into their catabolites, including phenolic acids. The absorption level of the resulting catabolites is, consequently, high enough to exert greater physiological actions than the parent flavonoids. The combination of quercetin-3-glucoside or α-glucosyl rutin with fructooligosaccharide (FOS) increases the concentration of quercetin and *O*-methylated quercetin conjugates in rat plasma compared with the plasma concentrations in rats treated with quercetin-3-glucoside or α-glucosyl rutin alone [118,119]. This suggests that FOS may decrease the degradation of quercetin via the modification of the intestinal microbiota and therefore, increase the bioavailability of the parent compound, quercetin. Dietary fiber shows promise in modifying the bioavailability and physiological functions of polyphenols by changing components of the microbiota.

The hydrolysable tannin, ellagitannin, is an oligomer composed of gallic acid, ellagic acid, and glucose. Ellagic acid is detected in human plasma 40 min after pomegranate intake [107,120]. However, plasma ellagic acid is undetectable within 5 h after the intake, suggesting it is rapidly cleared. Urolithins, which are well-known active catabolites of ellagic acid, and their conjugates can be detected in plasma at 0.5 and 6 h (0.04 and 0.11 μM of urolithin A respectively), with continuous excretion into the urine for 48 h after pomegranate consumption in some, but not all, volunteers [107,120]. Therefore, ellagic acid, produced by pH-dependent hydrolysis of ellagitannins [121], may be absorbed into the bloodstream at the upper digestive tract and may also undergo sustainable absorption as its bacterial catabolites, urolithins, at the colon. Since urolithin A and its conjugates are detected in some mouse tissues, including colon, prostate, liver, and kidney, at 1 to 6 h after oral administration of urolithin A [32], the continuous circulation of urolithins may allow their accumulation in tissues, thus facilitating their physiological functions.

The physiological functions of polyphenol catabolites, such as anti-oxidative, anti-inflammatory, and anti-proliferation activities, may differ from the functions of their parent polyphenols. Quercetin catabolites, such as 3,4-dihydroxybenzoic acid, 3-methyoxy-4-hydroxybenzoic acid, 3,4-dihydoxyphenylacetic acid, and 3-(3,4-dihydroxyphenyl)propionic acid, show one-half to one-eighth lower anti-oxidative activity than that of quercetin [122].

In an in vitro proximal colon model, green tea, black tea, and citrus flavonoids containing flavan-3-ols, theaflavin, quercetin, rutin, and hesperidin were catabolized to 4-hydroxyphenylacetic acid, 3-methoxy-4-hydroxyphenylacetic acid, 3,4-dihydroxyphenylacetic acid, and other catabolites. The anti-proliferation effect of these phenolic acids on colon cancer cell lines was one-fifth of the effect of the corresponding parent flavonoids [123]. A mixture of phenolic acids containing 3-(4-hydroxy-3-methoxyphenyl)propionic acid, 4-hydroxy-3-methoxybenzoic acid, 3,4-dihydroxybenzoic acid, and 3-(3-hydroxyphenyl)propanoic acid, which are detected in human plasma after blueberry juice consumption, have no effect on Nrf2 activation in human umbilical vein endothelial cells [124]. Furthermore, the inhibitory effect of phenolic acids, including 3-hydroxyphenylacetic acid, 3,4-dihydroxyphenylacetic acid, 3-phenylpropionic acid, 3-(3,4-dihydroxyphenyl)propionic acid, resorcinol, and phloroglucinol, on nitric oxide production in lipopolysaccharide-activated macrophages is minimal compared to the effects of their parent compound [125]. However, procyanidin A2 and its microbial catabolite, 3-(4-hydroxyphenyl)propionic acid, inhibit oxidized LDL-induced foam cell formation, inflammatory responses, oxidative stress, and the expression of cholesterol efflux/influx-related genes to the same degree, when applied at the same concentration [126]. Interestingly, urolithins show higher anti-oxidative, anti-inflammatory, and anti-proliferation activities than ellagitannin and ellagic acid [32,127,128]. Taken together with the pharmacokinetic data, bacterial catabolites can exert their specific functions in the large intestine and bloodstream, although the physiological function of polyphenols may be weakened or altered by the catabolism of parent compounds.

### 4.2. Action of Polyphenols on Gut Microbiota

Polyphenols are known to exhibit growth-promoting effects, namely prebiotic actions, on intestinal bacteria. This subject has recently been summarized in other review papers [129,130,131,132]. Although the molecular mechanisms of these prebiotic effects have not been fully elucidated, they may be associated, in part, with their selective antimicrobial activity against pathogenic bacteria. Phenolic extracts obtained from eight berries containing anthocyanins, flavan-3-ols, B-type proanthocyanidins, and ellagitannins inhibit the growth of food-poisoning bacteria, such as *Salmonella* Typhimurium, *Listeria monocytogenes*, and *Staphylococcus aureus*, but do not affect the growth of the probiotic bacterium, *Lactobacillus rhamnosus* [133]. Pomegranate ellagitannins strongly suppress the growth of *Clostridium* bacteria and *S. aureus* [134]. Lactic acid bacteria are also slightly reduced by treatment with ellagitannin, while the growth of bifidobacteria is markedly enhanced. However, in batch culture of human feces, pomegranate ellagitannin, but not punicalagin, increases the number of *Bifidobacteria* spp. and *Lactobacillus-Enterococcus* group bacteria after 5 and 10 h of inoculation, respectively, with no effect on *Eubacterium rectale-Clostridium coccoides* group and *Clostridium histolyticum* group bacteria [135]. Tannic acid from grape seeds and pomace show potent growth-promoting effects on *Lactobacillus acidophilus* [136]. Mango peels containing gallotannins also show prebiotic effects on bifidobacteria and lactic acid bacteria [18,137]. However, phenolic acids derived from the colonic bacteria-dependent catabolism of flavonoids have low selectivity against probiotic and pathogenic bacteria [138]. An intervention trial of 10 healthy adult men (45–50 years) investigated changes in the components of gut microbiota [139]. Drinking red wine containing anthocyanins, flavan-3-ols, B-type proanthocyanidins, flavonols, stilbenes, and phenolic acids for 20 days was shown to increase the proportions of the phyla *Proteobacteria*, *Actinobacteria*, *Fusobacteria*, *Firmicutes*, and *Bacteroidetes* in feces compared with baseline measurements. Meanwhile, de-alcoholized red wine with the same polyphenol composition only increased the proportions of *Fusobacteria* and *Bacteroidetes* and therefore, reduced the *Firmicutes/Bacteroidetes* ratio compared with subjects drinking red wine. Subjects drinking gin for the same period, as a non-polyphenol control group, showed a reduction in the proportions of these phyla. Curcumin and resveratrol also decreased the *Firmicutes/Bacteroidetes* ratio and have anti-inflammation and anti-carcinogenesis effects by modifying colonic microbial ecology in animal experiments [140,141,142,143,144]. Furthermore, resveratrol is thought to inhibit the production of trimethylamine and hence, trimethylamine oxide, from choline via the remodeling of the gut microbe composition [144]. Human microbiota-associated (HMA) animals (also known as human flora-associated, HFA) are germ-free rodents inoculated with a human fecal microbiota in order to study the human gut microbiome. Green tea polyphenols reduced blood glucose level, lipid metabolism biomarkers, and the *Firmicutes/Bacteroidetes* ratio in HFA mice fed a high-fat diet [145,146].

*Akkermansia muciniphila* is a gram-negative bacterium, isolated as a novel mucin-degrading bacterium from human feces [147]. Both live and pasteurized *A. muciniphila* are able to improve metabolic function in mice fed a high-fat diet [148]. This improvement is partly explained by the fact that an outer membrane protein, Amuc_1100, enhances glucose metabolism and gut barrier function by activating insulin and toll-like receptor 2 signaling [148]. The microbiome of mice on a high-fat diet show that an abundance of *A. muciniphila* is positively correlated with fatty acid oxidation and the browning of white adipocytes, but is negatively correlated with inflammation and metabolic syndrome makers [149]. This finding strongly suggests that *A. muciniphila* is a promising beneficial bacterium to prevent the development of metabolic syndrome. Interestingly, Colombian type 2 diabetes patients taking metformin have a higher abundance of *A. muciniphila* and SCFA-producing bacteria, such as *Butyrivibri*, *Bifidobacteria*, and *Megasphaera*, compared with those who do not take this drug [150]. Concord grape polyphenols, which appear to be composed of anthocyanidins, B-type proanthocyanidins, and flavan-3-ols, suppress inflammation in the intestinal tract and metabolic abnormalities and increase gut barrier integrity [20]. Cranberry extracts containing flavonols, anthocyanins, and proanthocyanidins also improve glucose and lipid homeostasis in mice fed high-fat and high-sucrose diets [22]. Oligomeric (monomer to tetramer) and polymeric (>pentamer) proanthocyanidins from apples suppress the abnormalities in both glucose and lipid homeostasis by modifying the expression of genes associated with inflammation, lipid metabolism, and gut epithelial tight junctions. However, there are some differences in efficiency between the oligomers and polymers [21]. More interestingly, only polymeric proanthocyanidins tend to decrease the *Firmicutes/Bacteroidetes* ratio and increase the relative abundance of *A. muciniphila* and *Verrucomicrobia* in the cecum of mice on a high-fat/high-sucrose diet. Although the role of *A. muciniphila* and *Verrucomicrobia* in the amelioration of metabolic abnormalities by these polyphenols needs to be fully investigated, the oligomeric and polymeric proanthocyanidins may exert these effects through a variety of molecular mechanisms.

Adhesion to the intestinal epithelia and subsequent colonization are thought to be important criteria for probiotic bacteria to exert their physiological effects on human health [151]. Epigallocatechin and procyanidin B1 and B2 increase the adhesion of lactic acid bacteria to intestinal epithelial cells in vitro, while some types of flavan-3-ols inhibit this adhesion [152]. Apple extracts containing procyanidin B2 and chlorogenic acid increase adhesion to intestinal epithelial cells in vitro. Quercetin and its glycosides show the most potent activity to enhance bacterial adhesion [153].

SCFA including acetate, propionate, and butyrate, are important metabolites from the action of colonic bacteria on indigestible carbohydrates. They play a role as physiological mediators by providing an energy source and acting as immunomodulators and metabolic regulators [154]. Rutin and some phenolic acids increase total SCFA levels and the ratio of acetate among SCFA in a multi-reactor gastrointestinal model [155]. The intake of a grapefruit extract, containing hesperidin and naringin, increases the wet weight of cecum digesta, and hence total SCFA content, in rats, while the combination of the extract with inulin weakened this effect [156]. Apple polyphenols containing epicatechin, procyanidins, and chlorogenic acid also increase rat cecum size and SCFA concentration in the cecum, and the combination of apple polyphenols with apple pectin shows an additive effect [157]. Inoculation with pomegranate ellagitannin increases the concentration of SCFAs in human fecal batch culture, while punicalagin has no such effect [135]. However, ellagitannin shows contradictory results for its combinational effect with FOS. Dietary FOS shows beneficial effects on pH, SCFA production, and bacterial enzymatic activity in the cecum. Different studies have shown that ellagitannin either interfered with or enhanced these positive effects of FOS [158,159]. Anthocyanins and phenolic acids may enhance SCFA production in the cecum of animals after FOS ingestion [118,159]. Flavonoids and phenolic acids, which promote the growth of SCFA-producing bacteria, including *Bifidobacterium spp.* and *A. muciniphilla* as mentioned above, may enhance the production of SCFAs. In addition, epigallocatechin-3-gallate, but not quercetin, stimulates the in vitro production of acetate and lactate by *Bifidobacterium adolescentis*, without growth-promoting effects [160], suggesting that flavonoids may be able to induce the production of SCFAs by colonic bacteria via activation of their metabolic function.

Culture supernatants of bifidobacteria and lactic acid bacteria are known to have anti-inflammatory activity [161,162,163], but both the active compounds and the mechanism of this action has not been fully demonstrated. When probiotic bacteria and polyphenols were anaerobically incubated for 3 h, a culture supernatant showed increased anti-inflammatory activity against lipopolysaccharide-stimulated macrophages [125]. Among 60 phytochemicals, quercetin, epigallocatechin-3-gallate, and some flavonoids increased the anti-inflammatory effects of *B. adolescentis* culture supernatant on nitric oxide production in activated macrophages in vitro [125,160]. On the other hand, these polyphenols were not able to potentiate the anti-inflammatory activity of lactic acid bacteria. Phenolic acids, which are assumed to be catabolites of these polyphenols, and the bifidobacteria metabolites, acetate and lactate, have no such effect [125,164], suggesting that these polyphenols may make *B. adolescentis* produce unknown active compounds. Interestingly, stearic acid has been tentatively identified as the anti-inflammatory compound obtained from the culture supernatant of quercetin-treated bifidobacteria [164]. Taken together, these findings suggest that polyphenols reaching the large intestine may not only be catabolized to small phenolic acids, but also elicit potentially beneficial effects of intestinal probiotic bacteria.

## 5. Future Directions

In recent years, metagenomic analysis of intestinal microbiota have provided a breakthrough in our understanding of the etiology of digestive diseases. Moreover, it is now recognized that changes in the intestinal microbiota affect the onset of not only digestive diseases, but also diabetes, atherosclerosis, neuropsychiatric diseases, and other systemic diseases [165]. However, the gut is an immune organ, in which more than half of all immune cells within the body are concentrated. The immune system in the gut affects the progression of obesity, diabetes, food allergies, and inflammatory bowel disease [166,167]. It is, therefore, of much interest to understand the relationship between the intestinal immune system and the intestinal microbiota. In this context, it is essential to focus research on elucidating the physiological function of polyphenols in the gut and their effects on the intestinal microbiota. In other words, microbiota should be taken into consideration when discussing the health effects of dietary polyphenols. It is, therefore, necessary to fully clarify the process of microbiota-dependent catabolism of dietary polyphenols in the large intestine. The effect of polyphenols on microbial behavior should also be evaluated in detail. As a whole, the relationship between dietary polyphenols and the intestinal microbiota is still obscure and unexplored. The intestinal microbiota is quite diverse among individuals within and between different ethnic groups. Therefore, the relationship between dietary polyphenols and microbiota is also diverse and profoundly complicated. To clarify the function of polyphenols in the absence of this confounding diversity, innovative in vitro experimental systems need to be established to replace rodent models, which have different microbiota to humans.

Finally, it should be noted that the role of microbiota in the brain–gut interaction is a future trend of polyphenol research. The intestinal microbiota is known to contribute to the development of reactions of the hypothalamic-pituitary-adrenal axis, which comprises major emotional reactions [25,168]. Microbiota is apparently recognized as a key factor in determining the stress responses and behavioral characteristics of the host. Successive intake of polyphenols may affect mental stress and behavior through the modification of intestinal microbiota. This is a subject on which future research should focus.

## 6. Conclusions

Polyphenols are composed of various subgroups whose basic structures differ for individual compounds. They can be divided into monomeric polyphenols and oligomeric tannins. After oral ingestion, some of the monomeric polyphenols and oligomeric tannins are absorbed, with or without deglucosylation, and enter into the blood circulation by crossing the epithelial cells in the small intestine. However, most polyphenols are transported into the large intestine, where intestinal bacteria catabolize them to chain fission products and/or monomeric catechins. Hydrolysable tannins, such as gallotannins and ellagitannins, generate gallic acid, ellagic acid, and their catabolites in the gut. Thereafter, these catabolites are excreted into the feces, but some of may be absorbed into the body through epithelial cells in the bowel. Polyphenols can be converted to various catabolites by the action of hydrolase and dioxygenase present in the microbiota. These catabolites may be non-negligible contributors to the health effects of dietary polyphenols. Furthermore, it is apparent that polyphenols, especially proanthocyanidins, have suppressive effects on the progress of life-style related diseases, such as diabetes, by modifying the patterns of the intestinal microbiota.

The intake of polyphenols improves the health effects of the intestinal microbiota by activating SCFA excretion, intestinal immune function, and other physiological processes. The microbiota-dependent effects of polyphenols may to be applied practically to the health food or supplement industries. For this purpose, further research is necessary to analyze the catabolic reactions of polyphenols and their reaction products, and to determine the mechanisms of action of these compounds on the intestinal microbiota.

## Figures and Tables

**Table 1:** Pharmacokinetic properties of flavonoid catabolites in human trials.

Samples	Subjects (Gender; Age; BMI)	Detected Compounds	C*_max1_* (M)	T*_max1_* (hours)	C*_max2_* (M)	T*_max2_* (hours)	AUC*_0-last_* (M·h)	T_1/2_ (h)	Refs
Blood orange juice (71 mg cyanidin glucosides)	Healthy men & women; 20–24; 21–31	Cyanidin-3-glucoside	0.0019	0.5			0.007 ^ac^		116
		3,4-Dihydroxybenzoic acid	0.492	2			11 ^ac^		
Cranberry juice cocktail (188.5 mg phenolics)	Healthy nonsmoking men & postmenopausal women; 50–70; 18.5–29.9	Quercetin	1.1	1.4	1.5	7.8	9.9 ^bc^		117
		Epicatechin	0.082	2.6	0.079	8.1	0.586 ^bc^		
		Myricetin	0.068	1.7	0.05	7.8	0.319 ^bc^		
		Isorhamnetin	0.099	0.5	0.153	7.3	0.267 ^bc^		
		Peonidin glycosides	0.00017–0.0021	0.9–4.7			0.00038–0.0053 ^bc^		
		Cyanidin glycosides	0.00019–0.00073	1.7–3.3			0.000092–0.0033 ^bc^		
		3,4-Dihydroxybenzoic acid	10	8.8			91 ^bc^		
		4-Hydroxy-3-methoxybenzoic acid	12	0.7	11	6.1	16 ^bc^		
		4-Hydroxybenzoic acid	1.4	0.8	1.4	7.2	9.9 ^bc^		
		4-Hydroxyphenylacetic acid	0.613	1.5	0.783	7.8	2.3 ^bc^		
		3,4-Dihydroxyphenylacetic acid	0.083	8.4			0.348 ^bc^		
[2-^14^C](−)-Epicatechin (207 mmoles)	Healthy men; 31 ± 3; 24.5 ± 3.3	Epicatechin conjugates ^g^	1.223	1			4.943 ^b^	1.9	50
		5-(3′,4′-Dihydroxyphenyl)-γ-valerolactone	0.272 ^d^	6.4			7.595 ^bd^	6.3	
			0.177 ^e^	6.1			3.237 ^be^	4.4	
			0.039 ^f^	5.5			1.017 ^bf^	6.4	
		5-(3-Hydroxyphenyl)-4-hydroxyvaleric acid	0.056 ^d^	5.9			1.492 ^bd^	7.6	
		5-(3,4-Dihydroxyphenyl)-4-hydroxyvaleric acid	0.054 ^e^	4.9			0.835 ^be^	6.5	
Pomegranate juice (318 mg of punicalagin; 12 mg of ellagic acid)	Healthy men & women; 32.6 ± 10.2; 21.3 ± 1.4	Ellagic acid	0.06	0.98			0.17 ^a^	0.71	107
Pomegranate juice (857 mg of gallic acid equivalent)	Healthy men & women; 29.7 ± 8.3; 24.1 ± 3.6	Ellagic acid	0.06	0.65			0.14 ^a^	1.14	120

^a^ 6 h; ^b^ 24 h; ^c^ Unit is converted based on the data of the references; ^d^ sulfate conjugate; ^e^ glucuronide conjugates; ^f^ sulfate and glucuronide conjugate; ^g^ sum of sulfate, glucuronide, and/or methylated conjugates.
